# Association of Immune Checkpoint Inhibitor Therapy With Survival in Patients With Cancers With *MUC16* Variants

**DOI:** 10.1001/jamanetworkopen.2020.5837

**Published:** 2020-06-12

**Authors:** Yunfang Yu, Dagui Lin, Anlin Li, Yongjian Chen, Qiyun Ou, Hai Hu, Herui Yao

**Affiliations:** 1 Guangdong Provincial Key Laboratory of Malignant Tumor Epigenetics and Gene Regulation, Department of Medical Oncology, Phase I Clinical Trial Centre, Sun Yat-sen Memorial Hospital, Sun Yat-sen University, Guangzhou, China; 2 Department of Colorectal Surgery, Sun Yat-sen University Cancer Center, Guangzhou, China; 3 Guangdong Medical University, Zhanjiang, China; 4 Department of Medical Oncology, The Third Affiliated Hospital of Sun Yat-sen University, Guangzhou, China

## Abstract

This cohort study investigates whether *MUC16* variation could be a useful biomarker for immune checkpoint inhibitor (ICI) therapy.

## Introduction

We and others have demonstrated that patients with cancer with a high tumor variation burden have derived encouraging benefits from immune checkpoint inhibitor (ICI) therapy.^1,2^ The oncogene *MUC16* (OMIM 606154) encodes cancer antigen 125, which has shown robust prognostic ability as well as critical involvement in the regulation of tumor variation burden and immune cellular dysfunction and resistance.^3,4^ However, there is no current clinical evidence of the association of *MUC16* variation with ICI therapy benefit. This comprehensive pancancer cohort study investigates whether *MUC16* variation could be a useful biomarker for ICI therapy.

## Methods

The study protocol was approved by the ethics committee of the Sun Yat-sen Memorial Hospital of Sun Yat-sen University. The requirement for informed consent of study participants was waived because the human data were obtained from publicly available data sets. This study followed the Strengthening the Reporting of Observational Studies in Epidemiology (STROBE) reporting guidelines.

We collected clinical and *MUC16* nonsynonymous variant data of 2129 patients treated with ICI and 10 812 patients treated without ICI from cBioPortal, PubMed, and The Cancer Genome Atlas. The median (interquartile range) follow-up of included patients was 21.0 (10.8-40.0) months. Overall survival (OS) and progression-free survival (PFS) were the primary outcomes, which were computed using the Kaplan Meier method and assessed with the log-rank test. The hazard ratios (HRs) were calculated by the Cox regression model. The tumor variant burden in *MUC16* wild-type vs variant tumors were compared with Wilcoxon rank sum tests. All analyses were performed from September 18 to October 1, 2019, using R version 3.4.4 (R Project for Statistical Computing). Statistical significance was set at *P* < .05, and all tests were 2-tailed.

## Results

**Figure 1.**
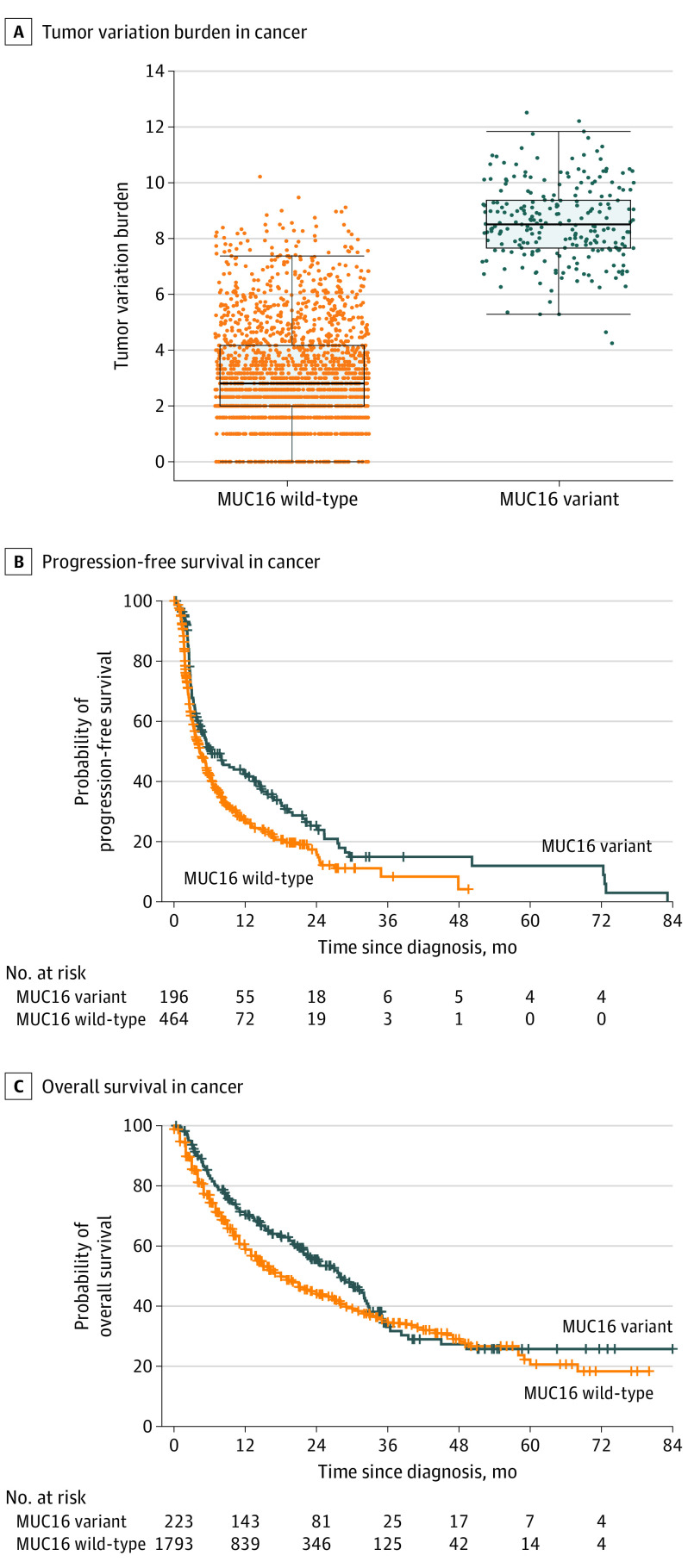
Association of *MUC16* Variation With Tumor Variant Burden and Survival With Immunotherapy in Pancancer A, The dots indicate the level of tumor variant burden for each patient. The box covers the interquartile range, with the upper and lower horizontal lines indicating upper and lower quartiles, respectively. The horizontal line inside the box indicates the median. The ends of the two lines outside the box indicate the highest and lowest data points, and the dots outside of these indicate outliers. B and C, A total of 2129 patients treated with ICI therapy were included in our pancancer analysis, of whom 675 (31.7%) had available data for progression-free survival (B) and 2016 (94.7%) had available data for overall survival (C). The crossing whiskers indicate censoring of data.

A total of 2129 patients who received ICI therapy were included (252 [11.8%] with *MUC16* variant; median [interquartile range] age, 63.0 [54.0-71.0] years; 1255 [58.9%] men), of whom 595 (28.0%) had melanoma, 510 (24.0%) had non–small cell lung cancer (NSCLC), and 1024 (48.1%) had 12 other cancer types. The median (interquartile range) tumor variation burden was significantly higher in the group with *MUC16* variant tumors than in the group with *MUC16* wild-type tumors (8.5 [7.7-9.4] vs 2.8 [2.0-4.2]; *P* < .001) (Figure 1A). Compared with patients with *MUC16* wild-type tumors, those with *MUC16* variant tumors had significantly longer PFS (HR, 0.70; 95% CI, 0.57-0.87; *P* < .001) (Figure 1B) and OS (HR, 0.79; 95% CI, 0.65-0.96; *P* = .02) (Figure 1C). However, among 10 812 patients with any cancer (2081 [19.2%] with *MUC16* variant) who did not receive ICI therapy, there was no difference in OS between *MUC16* variant and wild-type groups (HR, 1.06; 95% CI, 0.97-1.15; *P* = .19).

**Figure 2.**
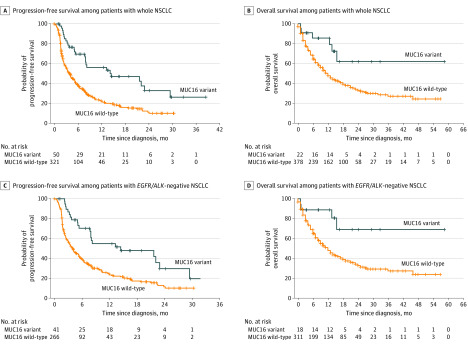
Association of *MUC16* Variant With Survival With Immunotherapy in Non–Small Cell Lung Cancer (NSCLC) Of 50 patients with NSCLC who were treated with ICI therapy, 22 (44.0%) had available data for overall survival and 50 had available data for progression-free survival. Among 378 patients with NSCLC who had *MUC16* wild-type tumors, 321 patients (84.9%) had available data for overall survival and 321 (84.9%) had available data for progression-free survival. The crossing whiskers indicate censoring of data.

Next, we found the clinical usefulness of *MUC16* variant status was most prominent in patients with NSCLC who received ICI therapy. Of 510 patients with NSCLC, 50 (9.8%) had *MUC16* variant tumors and, compared with patients with *MUC16 *wild-type tumors, had higher median (interquartile range) tumor variant burden (8.3 [7.5-9.3] vs 3.2 [2.3-4.4]; *P* < .001) and longer PFS (HR, 0.42; 95% CI, 0.28-0.64; *P* < .001) (Figure 2A) and OS (HR, 0.38; 95% CI, 0.17-0.86; *P* = .02) (Figure 2B). Moreover, among patients with *EGFR* (OMIM 131550)/*ALK* (OMIM 105590)–negative NSCLC, *MUC16* variation was associated with longer PFS (HR, 0.45; 95% CI, 0.29-0.70; *P* < .001) (Figure 2C) and OS (HR, 0.30; 95% CI, 0.11-0.80; *P* = .01) (Figure 2D). We found a significant PFS improvement in *MUC16* variant vs wild-type tumors among patients with *EGFR* or *ALK* variant NSCLC (HR, 0.32; 95% CI 0.11-0.92; *P* = .03). However, among patients receiving ICI therapy who had cancers other than NSCLC, we found no significant difference in OS (HR, 0.88; 95% CI, 0.72-1.07; *P* = .24) or PFS (HR, 1.08; 95% CI, 0.80-1.46; *P* = .60) between *MUC16* variant and wild-type tumors. We also assessed 481 patients with NSCLC (192 [40.0%] with *MUC16* variant) who did not receive ICI therapy and found no difference in OS between *MUC16* variant and wild-type tumors (HR, 0.94; 95% CI, 0.71-1.25; *P* = .66).

## Discussion

Using a large data set, we conducted the first study, to our knowledge, to report that *MUC16* could be a clinically meaningful biomarker for ICI therapy. The tumor variant burden is an important determinant of tumor antigenicity, and a gene variation is essential if it contributes greatly to affect the whole tumor variation profile.^5^ We found a significant association of *MUC16* variation with elevated tumor variant burden and prolonged PFS and OS during ICI treatment in pancancer and specifically in NSCLC, suggesting that *MUC16* might be an important component of the immunogenetic landscape and should be integrated into multiomics for precise selection of patients to receive ICI.

Furthermore, *MUC16* could be a potential therapeutic target to enhance ICI treatment outcomes. Adoptive transfer of *MUC16*-targeted T cells has resulted in powerful antitumor activity in vivo.^6^ It is conceivable that concurrently targeting *MUC16* and ICs might provide synergistic immune response. The study limitations include potential random variability in the context of an exploratory analysis contributed by NSCLC and our inability to clarify the mechanisms underlying the interaction between *MUC16* variation and ICI. Future prospective trials with a larger sample size and a longer follow-up period are needed to validate the pancancer applicability of *MUC16* variation status and characterize how *MUC16* variations interact with the immune system to affect the benefit of ICI therapy.
